# The Concept of Folic Acid in Health and Disease

**DOI:** 10.3390/molecules26123731

**Published:** 2021-06-18

**Authors:** Yulia Shulpekova, Vladimir Nechaev, Svetlana Kardasheva, Alla Sedova, Anastasia Kurbatova, Elena Bueverova, Arthur Kopylov, Kristina Malsagova, Jabulani Clement Dlamini, Vladimir Ivashkin

**Affiliations:** 1 Department of Internal Diseases Propedeutics, Sechenov University, 119121 Moscow, Russia; shulpekova_yu_o@staff.sechenov.ru (Y.S.); nechaev_v_m@staff.sechenov.ru (V.N.); kardasheva_s_s@staff.sechenov.ru (S.K.); sedova_a_v@staff.sechenov.ru (A.S.); kurbatova_a_a@staff.sechenov.ru (A.K.); bueverova_e_l@staff.sechenov.ru (E.B.); ivashkin_v_t@staff.sechenov.ru (V.I.); 2 Biobanking Group, Branch of Institute of Biomedical Chemistry “Scientific and Education Center”, 119121 Moscow, Russia; a.t.kopylov@gmail.com; 3 Institute of Clinical Medicine, Sechenov University, 119121 Moscow, Russia; jshulpekova@gmail.com

**Keywords:** folic acid, folate transporters, antifolates, folate overload

## Abstract

Folates have a pterine core structure and high metabolic activity due to their ability to accept electrons and react with O-, S-, N-, C-bounds. Folates play a role as cofactors in essential one-carbon pathways donating methyl-groups to choline phospholipids, creatine, epinephrine, DNA. Compounds similar to folates are ubiquitous and have been found in different animals, plants, and microorganisms. Folates enter the body from the diet and are also synthesized by intestinal bacteria with consequent adsorption from the colon. Three types of folate and antifolate cellular transporters have been found, differing in tissue localization, substrate affinity, type of transferring, and optimal pH for function. Laboratory criteria of folate deficiency are accepted by WHO. Severe folate deficiencies, manifesting in early life, are seen in hereditary folate malabsorption and cerebral folate deficiency. Acquired folate deficiency is quite common and is associated with poor diet and malabsorption, alcohol consumption, obesity, and kidney failure. Given the observational data that folates have a protective effect against neural tube defects, ischemic events, and cancer, food folic acid fortification was introduced in many countries. However, high physiological folate concentrations and folate overload may increase the risk of impaired brain development in embryogenesis and possess a growth advantage for precancerous altered cells.

## 1. Introduction

The discovery of folic acid is the result of hard work of Lucy Wills, a medical researcher graduating from Cambridge University with the degree of botanist and geologist. In 1930, she worked in India, having been especially interested in the problem of severe anemia in pregnant poor textile workers. Wills demonstrated amazing accuracy and insistence in her investigation excluding the infectious and parasitic nature of anemia and having come to the conclusion that it was linked to bad monotonous nutrition. After successful studies in rats, Wills suggested the use of special liver supplements and spreads made from brewer’s yeast in risk groups. Unknown substances possessing antianemic action together with improving the pregnancy outcomes at first was designated as “the Wills Factor”. Over time, other names were applied for this essential substance—vitamin M (“necessary for normal hemopoiesis in monkey”), vitamin B_c_ (“required for chicken growth”), Lactobacillus casei growth factor (“supporting Lactobacillus proliferation”), and vitamin B_9_. In 1941 folic acid was isolated from spinach (“folic” originates from Latin “folium” translating as “a leaf”) [1]. The cycle of industrial synthesis of folic acid was developed in 1945. Near that moment, Spies demonstrated the ability of folic acid to produce a prompt hematologic response in many cases of macrocytic anemia except those assessed to be “pernicious” [1].

**Figure 1:**
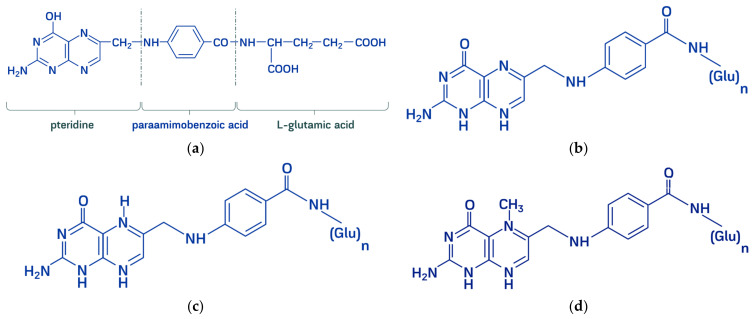
Chemical structure of (**a**) folic acid and its metabolically active derivates: (**b**) dihydrofolate, (**c**) tetrahydrofolic acid, (**d**) 5-methyltetrahydrofolate.

The chemical formula of folic acid is C_19_H_19_N_7_O_6_. The core of the molecule consists of heterocyclic pterin structure, with a methyl group in the sixth position bound to para-aminobenzoic and glutamic acids so that folic acid presents pteroylglutamic acid (Figure 1). Pterin is composed from pyrimidine and pyrazine rings («pteridine») with substituting keto- and amino groups in the second and fourth positions. Aromatic heterocyclic structures provide an ability for reversible electron-accepting [2,3]. “Folates” is a generic term encompassing folic acid and its derivatives-dihydro-, tetrahydro-, methyl-, formyl-compounds possessing metabolic activity. All folates are inherently conjugated with para-aminobenzoyl-glutamate as mono-, di-, tri-, and polyglutamates [4].

As the other pterins, folates represent ubiquitous and ancient metabolically active molecules. Pterins derivates were first discovered as the pigments of butterfly wing (Greek πτερόν is translated as «a wing»). They are found in mammals, bacteria, blue-green algae, trypanosomes, plant chloroplasts acting as cofactors, intracellular signaling molecules, ultraviolet protectors, and fluorescent pigments [4]. Pterin derivates are important regulators of bacterial behavior. Pterin glycosides and nucleotides are incorporated in respiratory chain and cyanide metabolism (e.g., in *Bacillus subtilis, Pseudomonas* spp.), three-carbon cycle (e.g., in *Escherichia coli*). Molibdopterins are essential for function of bacterial sulfite oxidase, nitrate reductase, dimethylsulfoxide reductase, nutritional C1-carbon chains, and metabolism of aromatic amino acids [2,3]. Tetrahydromethanopterin and tetrahydrofolate are more specific for methane-producing *Archaea* and methylotrophic bacteria [5]. In vertebrates, unconjugated pterins (tetrahydrobiopterin and molybdopterin) act as cofactors of lipid oxidase, nitric oxide synthase, sulfite and aldehyde oxidases, xanthine dehydrogenase, and in aromatic amino acids hydroxylation and serotonin cascade [6].

Active folates are highly sensitive to oxygen, sun light, high temperature and easily oxidized releasing pteridine and para-aminobenzoyl-glutamic acid. An exception is 5-formyl-tetrahydrofolate which is quite stable. Oxidation is inhibited by antioxidant such as ascorbic acid. Folic acid form water-insoluble complex compounds with divalent metals (Cu^2+^, Fe^2+^, Co^2+^, etc.) [7]. In biological fluids (blood), folic acid forms stable adducts with these cations. Researchers suggest that folic acid is involved in the elimination of divalent cations from the body [8].

## 2. Natural Sources of Folates

As some vitamins, folates could not be synthesized in mammalian cells and are delivered from exogenous sources, namely foods and intestinal microbiota [9]. In foods polyglutamylated folic acid, tetrahydrofolate (THF), 5-methyl-THF and 5,10-formyl-THF are ubiquitously present [10]. Animal liver and kidney, mushrooms, spinach, yeast, green leaves, and grasses are richest in folates. Table 1 provides a list of the top 20 foods richest in folate according to the National Institute of Health, The Office of Dietary Supplements [11].

Plants, fungi, certain protozoa, and several *Archaea* and bacteria synthesize folates de novo through similar pathways [12]. Microbial folate biosynthesis involves 16 enzymatic steps [9]. The common precursor for pterin biosynthesis is GTP [2,6]. On the first steps phosphoenolpyruvate combines with dietary para-aminobenzoic acid, GTP is converted to dihydropterin pyrophosphate by microbial GTP-cyclohydrolase and autoxidation take place [13]. Then, para-aminobenzoic acid combines with dihydropterin pyrophosphate. Last steps involve glutamylation and reduction with formation of folic acid and highly bioavailable 5,10-methenyl-THF and 5-formyl-THF [9,10]. Para-aminobenzoic acid for synthesis comes from the diet or is produced by intestinal microbiota in «nutritional chains». Genes implicated in folate biosynthesis were found across 512 gastrointestinal microbial genomes in the phyla *Bacteroidetes*, *Fusobacteria*, *Proteobacteria*, *Actinobacteria*. 13% of the genomes contain all genes required for de novo folate synthesis, and 39%—for folate synthesis in the presence of preformed para-aminobenzoic acid. Increased expression of folate synthesis genes was shown in exponential phase of bacterial growth, and increased polyglutamylation takes place during stationary phase [9]. Folate synthesis is best-studied in *E. coli*, *Bifidobacterium* [10,12,14]. Among the most popular probiotic strains this ability was shown for *Bifidobacterium* (especially *B. adolescentis* and *B. pseudocatenulatum*) and *Lactobacillus plantarum* (in the presence of para-aminobenzoic acid). Notably, physiological and genome analysis shows wild-type *Lactobacillus* cannot synthesize folate [12]. Distinct enzymatic pathways are responsible for the synthesis of other pterin derivates important for microbial surveillance.

## 3. Intestinal Folate Absorption

Absorption of dietary folates takes place in duodenum and proximal jejunum. Absorption and utilization may depend on the diet and cooking that is not very well studied [7]. 

Preliminary step is hydrolysis of polyglutamated folates by glutamate carboxypeptidase II in the enterocyte brush border. Monoglutamates are then transported intracellularly via proton-coupled folate transporter. Hydrolysis and absorption are optimal at low pH which is provided by intestinal Na+/H+ exchangers. Pool of folates produced by the colonic bacteria typically exceeds their dietary intake. There is direct evidence that folates might be absorbed across the colon via reduced folate carrier and proton-coupled folate transporter. The rate of colonic absorption is relatively low. However, its real contribution may be rather significant due to long transit time and abundant folate production [12,15]. Monoglutamylated folates seem to be absorbed at the highest rate [15].

### 3.1. Folate Cellular Uptake

**Figure 2:**
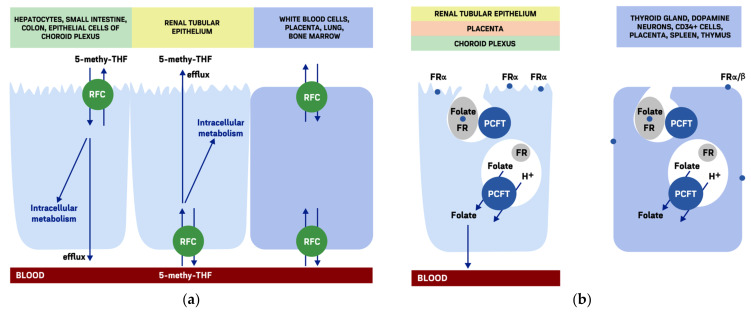

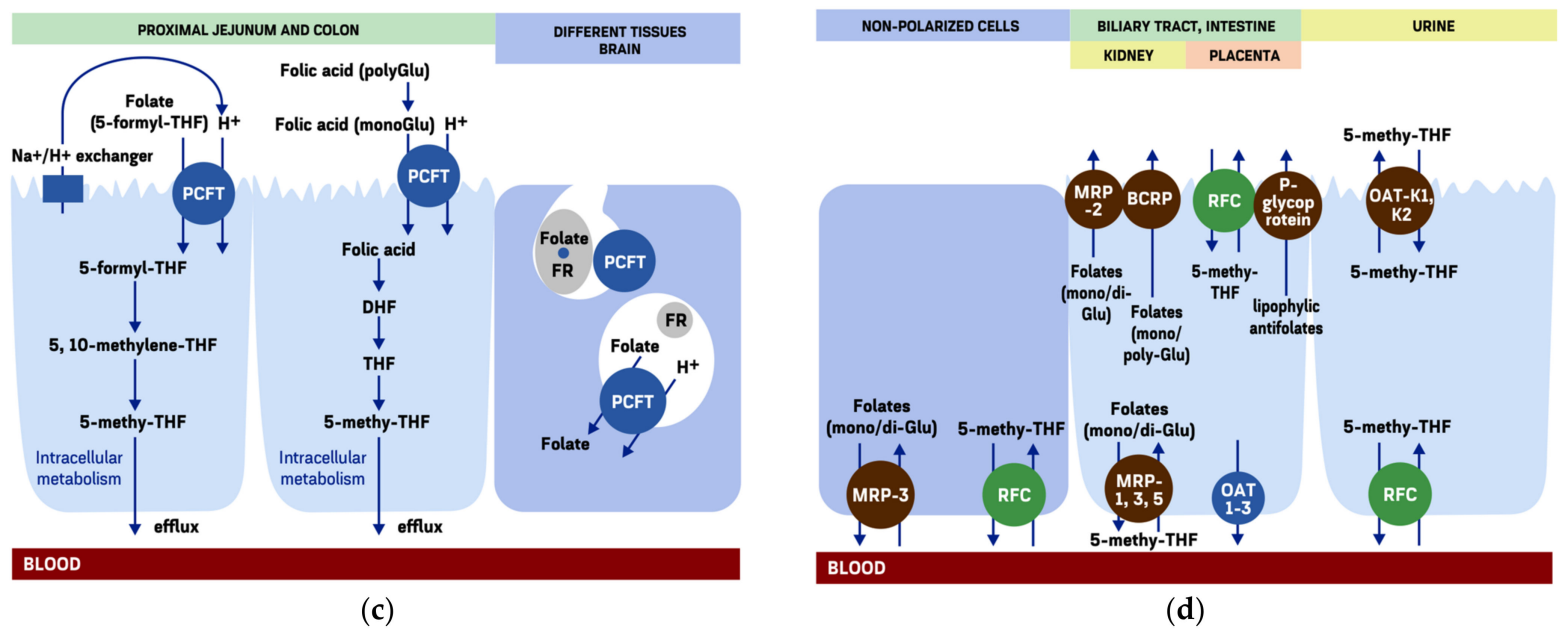
Schematic location and functioning of cellular folate transporters: (**a**) reduced folate carrier (RFC), (**b**) folate receptors (FR), (**c**) proton-coupled folate transporter (PCFT), (**d**) efflux pumps—multidrug resistance proteins (MRP), organic anion transporters (OAT), breast cancer resistance protein (BCRP). An apical surface of polarized epithelial cells is marked by irregular contour. In non-FRα-mediated transport, 5-methyl-THF rapidly converts into polyglutamates while in FRα-mediated transport it is rather moved transcellularly.

Folates are partially hydrophilic anions that do not easily diffuse across biological membranes [16]. Experimental data characterizing transport mechanisms were obtained using methotrexate as a chemical analogue of folic acid. 5-methyl-THF monoglutamate—circulating form of exogenous folate available for cellular uptake. In mammals 3 main transport systems are found with different tissue distribution, substrate affinity and specificity, and optimal operating pH (Figure 2). Homologic models of reduced folate carrier and proton-coupled folate transporter were developed based on the data coming from the studies of bacterial transporters GlpT and LacY [17].

### 3.2. Reduced Folate Carrier 

Reduced folate carrier (RFC) also designated as solute carrier family 19 member 1 (SLC19A1) seems to be the main pathway for folate delivery from the serum and extracellular space into most cells in physiological conditions. 

RFC is ubiquitous transporter found in placenta, white blood cells, kidney, lung, bone marrow; hepatocytes, small intestine and colon (apical membrane), epithelial cells of choroid plexus (apical membrane) the basolateral membrane of renal tubular epithelium [18]. In duodenum, proximal jejunum, and CNS the functional role of RFC is rather modest. In proximal small intestine folate transport from the lumen is mediated by proton-coupled folate transporter and from the serum to brain—by other two transporters which are expressed on the basolateral surface of the blood-brain-barrier contacting with endothelial cells blood vessels [18,19,20,21,22]. The. RFC is expressed ubiquitously with the maximal density in placenta playing a great role in transplacental folate transport. The lowest expression is in skeletal muscles [22]. Two other members of the SLC19 family (–A2 and –A3) providing thiamine transport and have a high homology to SLC19A1 [23]. 

RFC is a mammalian prototype of the major facilitator superfamily of transporters found in bacteria utilizing the electrochemical gradient of the target substrate (in “uniporters”), or second substrate (in “cotransporters”) [22,24,25,26]. RFC structure is very similar to that of GLUT1 glucose transporter [27,28]. RFC functions as a divalent anion exchanger and is neither directly linked to ATP hydrolysis nor is Na+/H+ exchange dependent [21]. RFC mediates vectorial folate transport depending on the transmembrane anion gradient provided mostly by organic phosphates (AMP, thiamine pyrophosphate) and folates [21,29]. Transmembrane organic anion gradient is being created by the independent exit pumps highly sensitive to cell energy balance [21].

In its quaternary structure of RFC protein is characterized by the presence of 12 transmembrane domains. N- and C- termini directed into cytoplasm [18,22,30]. Each RFC monomer functions as an independent transport unit [30]. A large loop connecting the sixth and seventh domains is directed into cytoplasm providing optimal space for transport between the adjacent domains [31]. Thiol-containing amino acids in 4th, 5th, 7th, 8th, 10th, and 11th domains are probably implicated in forming substrate binding pocket [32]. RFC doesn’t contain an ATP-binding region. 

Actually, RFC works as the bidirectional transporter for reduced folates, antifolates together with thiamine pyrophosphate also providing their export from the cell [33,34]. RCF possesses low affinity for folic acid (Michaelis constant (Km) 200–400 M) but ~50–100-fold higher for metabolically active reduced folates (Km 1–10 M) and “overall” antifolates (e.g., methotrexate, pralatrexate, pyrimethamine) providing competing transport of these substances [18,35]. Its activity is optimal at pH 7.4 which is physiological for serum. In weakly alkaline media folates are negatively charged due to complete ionization of two glutamate carboxyl groups. As pH is lowering, RFC-mediated transport falls becoming negligible at pH < 6.0–6.5 except the cases of RFC overexpression [36,37]. RFC activity significantly depends on transmembrane anion gradient and indirectly—on the cell energy balance. An abundance of organic and inorganic anions in extracellular compartment inhibits RFC-mediated folates influx, while depletion impairs folates efflux due to reduction of electrochemical driving force [22]. From the experiments with methotrexate, it became clear that the most potent intracellular anions inhibiting folate influx are 5-methyl-THF, thiamine pyrophosphate, AMP, ADP. As it was shown in L1210 cells, folates, para-aminobenzoyl-glutamate, smallest anions (Cl^-^, acetate, and lactate), divalent anions (phosphate and succinate), a few nucleotides (especially AMP), thiamine pyrophosphate are among the intracellular molecules stimulating RFC-mediated efflux [29]. Cellular ATP depletion provokes elevation of transmembrane folate gradient raising the impression of “more effective” RFC-mediated influx. That really may be explained by the inhibition of ATP-binding cassette proteins exporting folates from the cell, or the accumulation of AMP, with ADP potently inhibiting of RFC-mediated efflux [29,38,39]. 

Human RFC gene is located on chromosome 21q22.3 and contains 5 exons encoding a protein consisting of 591 amino acids [18,40]. Besides, there are up to 6 alternate non-coding regions preceded by a separate promoter. Regulation of RFC gene expression seems to be complicated and is not very well studied. Multiple RFC transcripts are identified due to alternative splicing of a single gene locus [41,42]. The alternative promoters transcribe sequences with generation up to 15 distinct 5′-untranslated regions fused to a common coding sequence. Alternative splicing for the non-coding exons A1/A2, A, B, and D 5′-untranslated region has been described, which is associated with profound decrease in RFC activity [22,25,43]. RFC-null mice showed very high embryonic lethality along with the absence of erythropoiesis in bone marrow, spleen, liver and lymphoid depletion in the spleen and thymus. Insufficient folate supplementation during gestation, live births are possible, but surviving pups die within two weeks due to inhibited hemopoiesis [44]. 

The degree of RFC transcription and regulatory region activation depends on the production of tissue-specific growth factors, degree of promoter methylation and chromatin structure [22,42]. 5′-untranslated regions of RFC are under strong post-transcriptional controls [22]. It was shown in HL-60 cells that RFC-mediated cellular transport decreases during cellular interphase (maturation stage) reflecting reduced synthetic activity [43,45]. Alternative splicing apparently plays an important role in the pathophysiology of folates deficiency and activity of antifolates [23]. The expression of folate transporter is sensitive to low-folate diet [46,47]. In breast cancer and T-cell leukemia cell lines, decrease in RFC and γ-glutamate hydrolase mRNA level was shown in short term (i.e., up to 7 days) folate deprivation. It was associated with consistent fall in methotrexate influx [32]. Adaptation to gradual lowering of extracellular folate is associated with their intracellular accumulation due to RFC gene amplification and overexpression, alternative splicing of truncated RFC, and exaggerated affinity to reduced folates [48]. The same compensatory changes were shown in small intestine and kidney in animal models [46]. Some factors produced by *Lactobacillus reuteri* may regulate RFC expression giving the reason to believe that other commensal bacteria may have the same effect [9]. RFC expression in choroid plexus may be significantly upregulated by calcitriol (1,25-dihydroxyvitamin D3), through the activation of nuclear vitamin D receptor. In the case of inactive folate receptor α and proton-coupled folate transporter, such stimulation of RFC presents the important alternative way for folates supplying to CNS [19,49,50]. As it is shown in diabetic retinopathy, RFC inactivity could be induced via oxidation of vicinal thiol groups by nitric oxide [48].

RFC polymorphism was studied mostly in clinical aspects: methotrexate resistance, hyperhomocysteinemia, risk of cancer, hereditary neural tube defects, and Down syndrome [16]. The most prevalent and best-studied polymorphisms are both in the promoter region (G80A) and in promoters A1/A2 and A [22]. G80A polymorphism may be associated with RFC downregulation leading to folate deficiency and methotrexate inefficiency although it is not confirmed in other studies [51,52,53]. In rheumatoid arthritis G80A polymorphism together with γ-glutamyl-hydrolase gene T401C polymorphism are associated with a significant decrease in intracellular methotrexate polyglutamate as compared with AA-genotype [54]. It may explain the association of G80A polymorphism with methotrexate toxicity showed in acute lymphoblastic leukemia [22]. In meta-analysis, no significant association between G80A polymorphism and overall risk of solid cancers, but in turn, some protective effect against digestive cancer risk were shown (GA-genotype vs. GG-genotype: OR = 0.89, 95% CI = 0.81–0.99, *p* = 0.030) [55]. In the same study, the probable pathogenetic role of G80A polymorphism in hematologic malignancy is underlined, although in meta-analysis, no association of G80A polymorphism with acute lymphoblastic leukemia was observed [55]. The authors suggest the direction of further research should focus on gene-gene and gene-environment interaction [56]. In many studies, G80A polymorphism was associated with the risk of fetal abnormalities. In 211 women having children with neural tube defects polymorphisms of three genes controlling folate metabolism (C677T—in methylenetetrahydrofolate reductase, C1561T—in glutamate carboxypeptidase II, and G80A—in RFC) was analyzed. When compared to the large control group, the clear relationship between the polymorphisms studied and the risk of neural tube defects for the whole group was not found. However, it was revealed that the homozygous variant of A80A of the RFC gene increased the risk of spina bifida by 2.55 times, and the risk of anencephaly by 3.28 times [57]. In a “case-control” study, RFC polymorphisms G80G increased the risk of developing neural tube defects by 2.35 times, and even more if it was associated with maternal polymorphism [58]. In the absence of folic supplementation during pregnancy, the risk of spina bifida in children with the GG-genotype increased by 2.4 times compared with AA-genotype. In folic supplementation, the GG-genotype carried 0.5-times increased risk [59]. Maternal G80A polymorphism and particularly GG-genotype might be associated with an increased risk of having a birth with Down syndrome as a consequence of abnormal DNA methylation leading to trisomy in chromosome 21 [16].

### 3.3. Folate Receptors 

Three Folate receptor (FR) isoforms are described in humans: α, β, γ [60,61]. FRα and FRβ are both glycoproteins anchored by glycosylphosphatidylinositol to cell membrane and having a mass of 38–40 kDa [61,62,63,64]. FRs are characterized by high affinity to folic acid and 5-methyl-THF (dissociation constant, Kd 1–10 nM), and lower to other folate derivates (Kd 10–300 nM). FRα and FRβ exhibit different stereometric specificity to 5-methyl-THF and 5-formyl-THF [65,66]. Isoforms also have some difference in terms of energy, ion, and pH-dependence being maximally active at pH ≥ 5.0 [67]. Normally FRs cellular expression may vary from minimal or even undetectable in some tissues to prominent in organs with high metabolic activity (i.e., placenta, thyroid gland, kidney, choroid plexus, midbrain dopamine neural progenitors, and nascent dopamine neurons) [68,69,70]. Minimal constituent expression of FRα/β is relatively tissue-specific [61,62]. FRα location is not strictly dependent on the cell polarity. Its noticeable expression is found in kidney on apical membrane of proximal tubular epithelial cells, choroid plexus, basolateral membrane of retinal pigment cells, in the uterus, and placenta [71,72,73,74]. High enough expression in choroid plexus helps to maintain the cerebrospinal fluid folate concentrations within relatively narrow limits; the same is true regarding placenta [49]. FRβ is expressed in CD34+ cells characterizing early hemopoiesis, placenta, spleen, and thymus [75,76,77,78]. FRγ by its nature is a secreted “signaling part” of FR protein lacking phosphatidylinositol anchoring it to the cell. Its physiological significance is not completely understood [79].

Cellular FRs location may be clustered in association with membrane invaginations—caveolae, characterized by the presence of lipoid rafts (organized combinations of glycosphingolipids, cholesterol and protein receptors). Caveolae provide sites for assembling cytoplasmic signaling molecules and have been implicated in cell adhesion and membrane trafficking [49,80]. FRs are associated with caveolin-1-containing microdomains playing the role of cellular growth regulators. Of great interest is the functional relation between caveolin-1 and FR-glycosylphosphatidylinositol anchor as the latter also may be involved in intracellular signaling [81]. However, FRs are not constitutively concentrated in caveolae, and in the absence of clustering stimulus may be diffusely distributed over the plasma membrane [80]. FR-mediated folate influx is not vectorial but involves mechanisms of receptor-dependent endocytosis [61,62,63,82,83]. The first pathway is known as potocytosis—receptor-mediated internalization in the sites of caveolae [84]. FR-folate interaction through phosphatidylinositol initiates membrane invagination and formation of an endosome. Subsequent acidification in this compartment results in dissociation of folate from the “FR-folate” complex (at pH∼6.5) with cytoplasmatic substrate export while FR returns on the cell surface [82,83,85]. Another pathway for “FR-folate” complex internalization is clathrin-coated pit endocytosis [83]. Internalization is dependent on cellular energy. Endosomal folate exporter functioning as anion exchanger in low pH is likely to be proton-coupled folate transporter [22,83,86,87]. FRα/β-mediated folate transport is not so efficient as RFC-mediated so FRs rather provide alternative pathway for folic acid [21,61,73,88]. However, in the case of impaired RFC function, FRs may present a significant transport route for folates and antifolates [80]. Low net FR recycling rate contributes to this [47]. FRα translocates to the nucleus where it acts as a transcription factor and upregulates Hes1, Oct4, Sox2, and Klf4 genes responsible for pluripotency [89].

As physiological folate concentration has been achieved, FR-mediated transport becomes markedly inhibited. That is however true only for FRs associated with caveolae. Cellular cholesterol depletion may predispose to raft domains dysfunction thereby impair the function of caveolae-associated FRs [49]. 

Overexpression of both FRα, FRβ has been shown in carcinomas of ovary, lung, breast, kidney, brain, endometrium and colon [65,90]. FRα overexpression is particularly characteristic for non-mucinous tumors of the ovary, uterus and cervix [73]. For ovarian carcinomas, FRα expression correlates with histologic grade and stage [91]. FRβ expression is especially characteristic for chronic myelogenous leukemia and for acute myelogenous leukemia [88]. It is likely that FRα overexpression and increased folate uptake confer a growth advantage for tumor cells, but in the other way might stimulate DNA reparation in the early stages of carcinogenesis [81]. High expression of FRβ on activated macrophages is seen in chronic inflammatory diseases such as rheumatoid arthritis, psoriasis, Crohn’s disease, and systemic lupus erythematosus [69]. FR-targeting is a perspective strategy for the diagnosis and treatment of cancers and immuno-mediated chronic inflammatory diseases. The use of tumor-targeted fluorescent folate dyes is suggested for malignant tissue marking in surgical resection [92]. FRα-agonistic medications might provide selective transport of active substance into the cell [90,93,94,95,96]. Functionalized magnetic Fe3O4 nanoparticles uptake through FR may be used for the targeted delivery and controlled release of water-insoluble chemotherapeutics agent [89]. FR-targeted liposomes containing photosensitizer meta-tetra(hydroxyphenyl)chlorin presents a novel delivery system for photodynamic anticancer therapy [97]. At the same time, FRβ-targeting is not suitable for diagnostic and therapeutic purposes in many cancers (colonic, ovarian, breast) due to its low expression in these malignancies [98]. FR-targeted imaging in positron emission tomography, γ-emitters and MRI is the promising approach helping to differentiate tumors with improved specificity and sensitivity [20,92].

FR α, β, and γ are homologous proteins encoded by three distinct genes located as a cluster on chromosome 11 [61,62]. Isoforms have highly conserved sequences (71–79%) in the open reading frame [81]. FRα gene contains 7 exons and 6 introns having complicated organization and transcription. The presence of multiple transcripts is possible due to the existence of 2 promoter regions and alternative splicing of exons 1–4 [81,99]. FRß and FRγ genes contain 5 exons, 4 introns and 1 promoter encoding a single transcript [81]. In cell culturess FRα activity may be upregulated by intracellular decline in metabolic active folates and excess of homocysteine [81,100,101]. Such feedback seems to be mediated by the interaction of homocysteine with FRα mRNA or hypomethylation of FRα gene which is rich in CpG bases highly subjected to methylation [81,100]. Thus, folate deficiency may act as epigenetic stimulus for FRα expression [81,102]. The same epigenetic mechanism may explain FRα overexpression in tumors [81]. Several studies have shown its negative regulation with estrogens and positive regulation with tamoxifen, dexamethasone, glucocorticoid receptor and retinoic acid [81,103,104]. It is likely that FRα is primarily under the influence of steroid hormones whilst FRß is regulated by retinoid compounds [105]. Increased FRs expression is associated with rearrangement in the FR promoter region leading to appearance of novel transcripts with enhanced stability [70]. FR overexpression probably contributes to antifolate resistance because of folates-antifolates competition in the internalization [49]. Many details of FR genes regulation remain to be unclear.

FRα gene function plays an essential role during embryonic development, but seem to be not so important in adult life except in folate transport to the CNS [49]. FRα-null mice are embryonic lethal in the absence of special folate supplementation during gestation [106]. FRβ-null mice don’t express pathological phenotype [106]. In human mutations in FRs genes appear to be infrequent [81]. Several silent mutations were found including those interfering with translation, membrane FRα binding and folate internalization and action of transcription factors. Later in life, these mild defects might predispose to hyperhomocysteinemia, increased renal folate clearance, insignificant serum folate decrease, etc. [81]. Cerebral folate deficiency (CFD) is an autosomal recessive disorder caused by biallelic pathogenic loss-of-function variants FRα gene leading to impaired folate transport from blood into the brain through the choroid plexus. Several families have been reported with this abnormality [49,106]. 

### 3.4. The Proton-Coupled Folate Transporter

The proton-coupled folate transporter (PCFT) also designated as solute carrier family 46 member 1 (SLC46A1), was described in 2006 [18]. In contrast to RFC it is characterized by high affinity to folates at low pH that is [18,36,106,107,108]. PCFT functions as a folate-proton symporter. More than two protons are co-transported with each folate bivalent anion to account for the positive charge of the PCFT-folate-proton complex [18]. Folate influx is noticeable at pH 5.5–7.3 becoming negligible and overcoming pH 7.4. Similar to RFC, PCFT is stereospecific for reduced folates having maximal affinity to 5-formyl-THF [107,109]. PCFT may be considered as a classical apical epithelial transporter providing absorptive function. High PCFT expression is found in enterocytes (maximally—in proximal jejunum and duodenum), renal tubules, hepatocytes, placenta, retina [107,108,110,111,112,113]. Lower levels are found in caecum and colon, testis, brain, lung, stomach and also in the heart and muscles although these tissues do not contain polarized cells [114]. In proximal jejunum where the maximal absorption of dietary folates occurs, apical brush border Na+/H+ exchangers generate pH of 5.8–6.0 which is optimal for PCFT function. PCFT is considered to play an essential role in intestinal folate absorption. PCFT is also expressed within FRs-containing endosomes and here provides folate export into the cytoplasm at pH ∼6.5 [85]. 

PCFT protein contains twelve transmembrane domains with C- and N-termini oriented to the cytoplasm and large glycosylated extracellular loop. It is energy-dependent, saturable and partially inhibited by ionophores [115]. 

SLC46A1 gene is located on chromosome 17q11p2. Loss-of-function missense mutation is associated with serious pathology known as “hereditary folate malabsorption (HFM)” [116,117]. HFM is an autosomal recessive pan-ethnic disorder; 38 affected families have been described in the world. Heterozygotes are symptom free.

In mice, in a folate-deficient diet, RFC and PCFT expression is increasing in small intestine [49,111]. Although underlying regulatory mechanisms are not well understood, it can be assumed that methylation of regulatory gene areas may be involved.

## 4. Extracellular Efflux 

Potential folate export routes are represented by RFC, P-glycoprotein, and multidrug resistance-associated proteins (MRP)-1-5 related to ATP-binding cassette (ABC) exporters and the breast cancer resistance protein (BCRP), which are widely expressed in mammalian cells [22,118,119,120,121,122,123,124]. ABC exporters provide unidirectional folate transport together with other anions including bilirubin, xenobiotics, and endogenous toxic metabolites, leukotrienes, glutathione, and some medications [30,124,125,126,127]. These transporters have low affinity and high capacity for folates/antifolates (K_m_s ∼ 0.2–2 mM). Long chain (*n* > 3) folate polyglutamylates cannot be extruded via these export pumps while short-chain derivates may be weak substrates for MRPs [122,128]. BCRP is capable of transporting mono- and polyglutamate folates and plays an important role in cellular folate homeostasis. As it was shown in MCF-7 breast cancer cells, BCRP downregulation, together with increased folylpoly-gamma-glutamate synthetase activity, appear to be crucial components of cellular adaptation to folate deficiency [129]. P-glycoprotein is capable to transport large lipophilic drugs [49]. P-glycoprotein, MRPs and BCRP are mostly expressed in tissues with “polarized” cells with clear differentiated apical and basolateral membranes [22]. MRP2 and P-glycoprotein are localized almost exclusively to the apical membrane of hepatocytes, enterocytes, renal proximal tubule epithelium, placentic syncytiotrophoblasts and might play a role in enterohepatic folate circulation [116,117]. MRP-1, -3-5 are mostly localized to the basolateral membrane [126]. MRPs activity is modulated by the agonists of several nuclear receptors (pregnane X receptor, constitutive androstane receptor, glucocorticoid receptor, *peroxisome proliferator-activated receptors*), and may significantly change under the influence of hepatotoxic substances, in cholestatic, inflammatory, and malignant diseases [126]. MRPs genes are rich in GC that may testify to the important role of methylation in the control of their activity [126]. Substances affecting cytochrome and leading to inhibition of ATP synthesis (e.g., polyphenoles) simultaneously impair MRPs function [130]. 

The members of solute carrier family 21 and 22 (SLC21 and SLC22) are also involved in folate influx and efflux [22]. Organic anion transporters K1, K2 (OAT-K1, -K2) expressed to the apical brush border of renal proximal tubular cells, mediate bidirectional transport of substrates including folates and methotrexate. OAT-K1, -K2 might play a role in excretion of hydrophobic toxic organic anions from tubular epithelial cells into urine [131]. OAT1-3 are expressed at the basolateral membrane of renal tubules and are also candidates for pumps mediating folate efflux [132].

## 5. Intracellular Transformations

In enterocytes and hepatocytes absorbed folic acid at first is reduced by dihydrofolate reductase to dihydrofolate (DHF) and tetrahydrofolate (THF). THF is converted to 5,10-methylene-THF by pyridoxine-dependent enzyme serine hydroxymethyltransferase and then is reduced to 5-methyl-THF by methylenetetrahydrofolate reductase (MTHFR). THF and 5-methyl-THF are the most biologically active folates playing essential role in cellular one-carbonic metabolism. 

From the enterocytes and hepatocytes 5-methyl-THF is transported through basolateral RFC and other efflux pumps to the circulation and then uptaken by RCF, FRs, PCFT in extrahepatic tissues. In the case of dihydrofolate reductase inactivity or overload, oxidized folic acid may appear in circulation [132]. Folate derivates can be efficiently retained in the cells only due to polyglutamylation which is provided by folylpoly-γ-glutamate synthetase adding glutamate units to γ-carboxyl residues [23]. Polyglutamates present higher affinity to folate-utilizing enzymes [133,134]. The most preferential substrate for folylpoly-γ-glutamate synthetase is THF. Thus, to facilitate polyglutamylation, 5-methyl-THF should be converted to THF by methionine synthase. Polyglutamylation rate is driven by intracellular concentration of folates/methotrexate and is ATP-dependent [135,136,137]. For folates that entered the cell through FR and RFC, the rate and extent of polyglutamylation are similar. Both receptors appear to deliver folate to the same intracellular compartment to the location of folylpoly-γ-glutamate synthetase [138]. As folates are also essential to mitochondrial metabolism, polyglutamates are transported as well into mitochondrial matrix [125]. 

### Folates in One-Carbon Pathway

**Figure 3:**
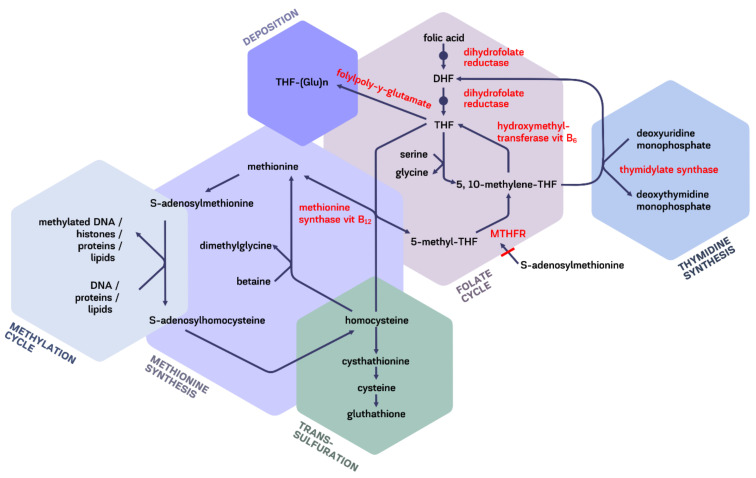
Cellular cycles involving folates. The names of enzymes are marked by red. Intracellular transformations of 5-methyl-THF may depend on the way of absorption. The steps where NAD^+^/NADH is involved are marked by blue circles. Adapted from [139].

One-carbon pathway is an essential biochemical cycle in which methyl groups are transferred from molecules-donors. It is closely related to thymidine and methionine synthesis, methylation of different molecules, trans-sulfuration (Figure 3). 

Folates are cofactors due to the presence of pterin core having π-electron-deficient properties and easily reacting with O-, S-, N-, C-bounds [140]. One-carbon pathway plays an important role in methylation of nucleic acids, histones, neurotransmitters, phospholipids, proteins, homocysteine remethylation and indirectly in glutathione reduction [141]. The metabolically active THF as an intermediate carrier of single carbon unit plays a central role. The most important sources of one-carbon groups are amino acids serine, glycine, histidine, tryptophane, and the “biosynthetic destinations”—purine bases, thymine and S-adenosylmethionine. Available methyl groups may be diverted from the DNA synthesis pathway toward the other cycles-methionine synthesis, trans-sulfuration, polyglutamate deposition, etc. [142]. Methylation cycle provides one-carbon transferring to choline phospholipids, creatine, epinephrine, DNA. DNA methylation is the basic mechanism for transmitting of DNA methylation patterns after DNA replication, epigenetic regulation with alternative gene splicing and tissue-specific gene expression, X-chromosome inactivation, imprinting [143]. This covalent DNA modification commonly occurs at cytosines within CpG-dinucleotides. DNA methylation issues are not completely understood. Periods of dynamic reprogramming of DNA methylation patterns during gametogenesis (still before the conception) and embryogenesis present a “windows of opportunity” for the influence of exogenous factors [143,144]. That is why it seems to be very important for the development of appropriate folic food fortification for women of child-bearing age. A pathological pattern of DNA methylation may be the cause of tumor suppressor gene silencing and chromosome instability and cancer [144]. Global methylation is usually assessed in peripheral blood cells or in the tissue sample. 

Trans-sulfuration is highly important for endogenous detoxication, glutathione synthesis which, in turn, support the extracellular toxins efflux. Thymidine synthesis provides the material for nucleinic acids formation [145].

## 6. Problem of Folate Deficiency and Oversupplementation 

Some inconsistences in definition of “low folate status” can be seen in different papers, where it may correspond to low blood folate concentration, low folate intake or the presence of macrocytic anemia. Laboratory criteria of folate deficiency according to WHO recommendations are presented in Table 1 and Table 2. Serum folate level varies with dietary intake throughout the day [146]. Therefore, a single quantitative measurement of folic acid in the blood cannot be used as a clinical criterion. However, repeated low serum folate levels in humans over several weeks indicate “low folate status”. Conversely, the content of folate in erythrocytes is slow to respond to changes in dietary folate intake and is an important clinical indicator [146].

Food Frequency Questionnaire (FFQ) may be used for assessment of total dietary folate equivalent [147]. Bioavailability of synthetic folic acid may be more predictable. Recommended daily dose may be expressed in μg of «food folate equivalent» where 1 μg of “food equivalent’ approximately corresponds to 0.6 μg of synthetic folic acid [148]. Recommended daily intake of folic acid according to the National Institute of Health (Dietary Supplement Fact Sheets) are presented in Table 2 [11].

Folate deficiency is estimated to be most prevalent vitamin deficiency over the world [146,149,150]. Folate deficiency is seen in up to 10% of the USA population. Combined deficiency of both vitamin B_12_ and folate is highly prevalent in Pakistan along with mild hyperhomocysteinemia and coronary artery disease [151,152]. Folate deficiency and associated metabolic disorders are probably the most actual for the elderly, who are not provided with appropriate nutrition and women of reproductive age who follow strict diets [148]. In the Taiwanese elderly population, hyperhomocysteinemia is seen in 23.4% of males and 11.2% of females and increased with age occurred only in those who had concurrent poor folate, vitamin B_6_, or B_12_ status. The strength of the association between vitamin B_12_ deficiency and hyperhomocysteinemia increased about three-fold when combined with low folate [148]. The systematic review of studies assessing folate deficiency in women of reproductive age in 39 countries shows its prevalence of >20% in countries with lower income economies and <5% in countries with higher income economies. A total of 11 surveys reported the prevalence of folate deficiency >40% in most countries [153]. More than half of German women of reproductive age do not consume sufficient dietary folate to achieve optimal serum or red blood cell folate concentrations necessary to prevent neural tube defects—spina bifida, spinal hernia, and anencephaly [154]. Younger maternal age, lower educational level, and lower family income are suggested predictors of low folate status [155,156]. High serum cotinine concentration as a marker of active and passive smoking is associated with increased risk of folate deficiency [155]. Supplementation of folate combined with mandatory fortification of foods has led to high levels of folic acid and related metabolites in women of childbearing age [157].

Based on the data showing potential role of folate in reduction of cardiovascular events, cancer and neural tube defects, the program of folate food fortification was initiated in USA in 1998 and later introduced in Canada, Chile, Israel [158,159,160,161]. Contemporary recommended supplementation for pregnant women is 400 μg, for breastfeeding women-500 μg, for others-400 μg of “food folate equivalent” daily [148]. Exceeding the recommended dietary allowance of folates may also have a negative effect on human health. Theoretically risk of adverse effects, such as progression of cancer, development of neuropathies due to masking the diagnosis of cobalamin, abnormal CNS development in embryos. Some results suggest that in heavy smokers, high folate levels add to the cancerogenic effect of smoking. The study from northern Poland included 132 lung cancer patients and 396 controls. The median cigarette pack-years of smoking among both cases and controls was 30.0. Serum folate concentration above the median (>17.5 nmol/L among the healthy controls) was associated with an increased lung cancer risk (OR 1.54, 95% CI 1.04–2.29, *p* = 0.031). An analogous trend was observed when the population was analyzed after subdivision according to RBC folate concentrations. In a subset of women, an increased risk of lung cancer was associated with the RFC gene A80A polymorphism (A80A versus G80G OR, 3.14; 95% CI, 1.32–7.46; *p* = 0.010) [162]. In a very elegant experiment with human keratinocytes immortalized by human papillomavirus HPV16 it was shown that folate depletion causes irreversible DNA damage, impairment of DNA repair fidelity, and unique chromosomal alterations. In repleted folate, state cells with anomalous phenotype underwent growth advantage. The authors underline the controversy of folate fortification programs especially in developing countries were human papillomavirus is highly prevalent [163]. At the same time, in dietary folate deficiency in Beige Nude XID mice, rapid transformation of differentiated organotypic raft-keratinocytes infected with human papillomavirus to cancer was found [164]. These data show the importance of previous folate status assessment. Theoretically carcinogenic effect of folic acid supplementation also may be related to methylation and silencing of CpG island–associated tumor suppressor genes observed in cancer. However, today, there are insufficient data to determine real effect of higher doses of folic acid at any particular genomic regions or specific tissue type [143]. In contrast, methyl donor-deficient diet (low in folic acid, choline, methionine, and vitamin B_12_) is associated with sustained tumor protection of colonic mucosa in *Apc*-mutant mice *(Apc^Δ14/+^)*. Approximately 100 metabolites affected by the methyl-deficient diet were identified in colonic samples: reduced methionine (−2.9-fold, *p* < 0.001) and betaine (−3.3-fold, *p* < 0.001), elevated homocysteine (110-fold, *p* < 0.001) with activation with trans-sulfuration [165]. High-physiological folic acid concentrations differently influence malignant and non-malignant colonic cells that is clearly demonstrated in cell lines HT29 and HCEC, respectively. While high-physiological FA concentration had no influence on promoter methylation, in the HT29 cell line, tumor-suppressive micro RNAs were predominantly downregulated and the expression of genes involved in chemotaxis and immunity was modulated. In non-malignant HCEC cells folic acid didn’t affect pro-inflammatory genes and cancer-associated microRNA expression [166]. Some studies demonstrated that DNA methylation may be considered as a biomarker of colorectal cancer and that methylation of DNA in the colonic tissues and leukocytes can be increased by supplementation with folic acid [143,167,168]. In the trial, examining the effect of folic acid supplementation (1 mg/day for three years) on site-specific DNA methylation (limited to two loci of estrogen receptor alpha and secreted frizzled related protein-1) no statistically significant associations of methylation at were found with hyperplasic polyps or adenomas [143,169]. Many types of cancers overexpress FRs. FRα may provide a nuclear signal for activation of genes responsible for cellular pluripotency, and its acetylation and phosphorylation favor its nuclear translocation in the presence of excessive folate [85]. This work delineates how high folate levels could cause dedifferentiation of already differentiated glial cells [85].

In a brilliant review highlighting the problem of probable maternal over supplementation during pregnancy, different problems related to neurological and metabolic abnormalities in offspring [157]. Special attention is paid to 8.2-fold increased incidence of autism probably associated with prenatal supplements containing over 1000 µg of folic acid and the percentage of pediatric vitamins containing any folic acid [170]. The greatest risk was observed in mothers with elevated levels of both vitamin B_12_ and folate, while homocysteine level and *MTHFR* genotype, did not predict the risk of autism spectrum disorders [171]. Many studies in vitro and in vivo confirm the potential risk of folate over supplementation showing abnormal development of hippocampus, cerebellum, behavioral changes, memory impairment, seizures, and high risk of obesity [157]. In the Indian population, higher maternal folate in pregnancy predicted higher adiposity and insulin resistance in children at the age of six, exaggerated by low maternal vitamin B_12_ [172]. The problem of optimal time and doses of folic acid supplementation in pregnancy remains really acute.

## 7. Laboratory Folate Metabolism Assessment 

Laboratory testing on the first step should involve evaluating serum/plasma folate level. 5-methyl-THF is the most prevalent physiological form of folate in systemic circulation. Fasting serum folate more specifically reflects folate level in tissues. One should take in account falsely normal results due to postprandial increase (within 2 h) in non-fasting samples. Falsely normal or elevated folate is typical of sample hemolysis or vitamin B_12_ deficiency in which folate to be “trapped” as 5-methyl-THF. To minimize the risk of mistake it is reasonable to repeat assessment of serum folate level throughout the month [173]. Erythrocytes accumulate folate only during erythropoiesis and their life expectancy is 120 days, so red blood cells folate content reflects folate status for the prior 3 months and is not affected by recent dietary intake. Recent blood transfusion can lead to inaccurate results [174]. There is a variety of methods for the assessment of serum or plasma and RBC folate concentrations—microbiological assay, competitive radioisotope method, binding and enzymatic or chemiluminescent assays, and LC-MS/MS [175]. Large enough differences in accuracy among these folate assays have been observed. Microbiological assay is based on the ability of Lactobacillus rhamnosus to grow in the presence of folate monoglutamate. This method is viewed by many researchers as the “gold standard” [173]. LC-MS/MS has been recommended to quantitate individual folate forms which may be useful for characteristics of metabolic alterations, e.g., in polymorphism in MTHFR (C677 > T). LC-MS/MS also helps to identify folic acid in serum if patient is using folic acid containing supplements [175]. In clinical practice automated immunoenzymatic assay is widely used [175]. The cutoffs of normal value were taken on the basis of the likelihood of macrocytic anemia development [150,173] (Table 3). Interpretation of the results sometimes may be difficult, so assessment of homocysteine level as a non-direct marker of folate deficiency may be used (Table 4) [173].

## 8. The Main Clinical Syndromes of Folate Deficiency

### 8.1. Hereditary Syndromes

Folate metabolism is essential for CNS development and functioning. FRα and PCFT are highly expressed in epithelial cells of choroid plexus (PCFT is likely to mediate folate transport from endosomes to the cytoplasm). Hence, functions FRα and PCFT are closely interrelated and inactivation of any of these receptors results in low folate concentration in liquor. ***CFD*** develops due to loss-of-function mutation of FRα gene. Intestinal folate absorption is not impaired in this case. 5-methyl-THF concentration in serum is within normal range and hemopoiesis is not impaired. 5-methyl-THF is decreased in cerebrospinal fluid and neurologic signs develop within several years after birth [49,106]. Such manifestations are rather nonspecific, including delayed psychomotor development, ataxia, tremor, chorea, and myoclonic seizures. In MRI signs of hypomyelination may be found. CFD also may develop due to production of blocking FRα autoantibodies which manifest by autism spectrum disorders regressing in course of treatment with folinic acid. In some cases of catatonic schizophrenia with positive FRα autoantibodies, regress of auditory hallucinations was shown due to the appointment folinic acid [1,176,177]. HFM is characterized by impaired intestinal folate absorption and insufficient transport into CNS due to loss-of-function of mutation of PCFR gene. Even parenteral folate supplying could not compensate its transport in CNS. Infants with HFM may be born with adequate stores of folate but being incapable to absorb them from breast milk or formula become folate-deficient [117]. Clinical signs usually develop in first several weeks of life and include poor feeding, normo/macrocytic anemia, pancytopenia, diarrhea, oral mucositis, hypoimmunoglobulinemia, opportunistic infections (most often Pneumocystis jirovecii pneumonia) and different non-specific neurologic manifestations—cognitive and motor impairment, behavioral disorders, ataxia, peripheral neuropathy, seizures [117]. In PCFT-null mice atrophy of all hematopoietic tissues (bone marrow, liver, spleen, thymus) develops [116]. Diagnosis of HFM is confirmed by low serum folate level unresponsive to oral folate load and decreased cerebrospinal fluid folate concentration (not changing even after correction of the serum folate concentration). It is very important to take into account family history of neurological pathology (e.g., seizures) in newborns [116]. Molecular genetic testing identifies biallelic pathogenic variants in SLC46A1 gene. Both CFD and HFM must be differentiated with vitamin B_12_ deficiency, inadequate dietary folate, intestinal disease associated with folate malabsorption, myeloproliferative diseases, X-linked severe combined immunodeficiency, methionine synthase deficiency, and rare mitochondrial disorders [116]. Early treatment with high doses of oral 5-formyltetrahydrofolate (folinic acid) per os or parenterally can obviate the manifestations both of CFD and HFM [116]. 

### 8.2. MTHFR Deficit

MTHFR activity is reduced in the common genetic polymorphism C677T. CT-variant of MTHFR gene increases the risk of low folate status and hyperhomocysteinemia. MTHFR TT-genotype has a prevalence up to 15–20% in some populations, and meta-analysis confirms its association with lowered serum folate, increased homocysteine and lack of the response to short-term folate supplementation [114,178]. TT-genotype carries elevated risk of thromboembolism (OR 1.2) and stroke (OR 1.26). Increased homocysteine level was found in homozygotes in polymorphism G80G in RFC gene and polymorphism T677T in MTHFR gene (*p* < 0.05 compared with the G80G/C677C and G80G/C677T—genotypes) [53]. The risk of neural tube defects seems to be increased in MTHFR gene C677T polymorphism but may also be influenced by MTHFR gene polymorphisms A1298C and methionine synthase reductase gene polymorphism A66G [179]. Homozygotes in A1298C-genotype have higher levels of global DNA methylation (*p* = 0.04) [147]. 

### 8.3. Folylpoly-γ-Glutamate Synthetase Deficit

This enzyme resides in both the cytoplasm and mitochondria. Knockout mice in folylpoly-γ-glutamate synthetase gene results in embryonic lethality [133]. In humans, the problem of hereditary folylpoly-γ-glutamate synthetase deficit is not studied.

### 8.4. Acquired Folate Deficiency

The most important causes of acquired folate deficiency are reduced intake, chronic alcohol consumption, diseases affecting the proximal small bowel (parasitic infestations, celiac disease, Crohn’s disease, etc.), increased demands in pregnancy, chronic hemolysis, intensive growth in puberty, and eczematous conditions [149,150,174]. Being toxic for mitochondria ethanol directly affects biological methylation reactions and inhibits synthesis of S-adenosylmethionine which is the primary methyl group donor [4]. Ethanol inhibits expression of RFC and PCFT, and intestinal and renal folate absorption [4,180]. Approximately 80% of chronic alcoholics admitted to a hospital may have low serum folate levels, in 44% corresponded to severely deficient range [150]. Alcohol decreases depot in the liver [181]. Alcohol consumption affects the functions folate-related genes and enzymes including major folate-metabolizing enzymes, aldehyde dehydrogenase 1 family members L1 and L2 (ALDH1L1 and ALDH1L2) and NAD^+^/NADH balance [181]. The problem of covert folate deficiency in liver and kidney diseases is not very well studied. The liver mediates folate extraction from portal blood through RFC, FRs, PCRF (although the expression of latter is relatively low). The liver seems to be a major storage of folate [49,81,180]. Folate efflux into the bile is mediated by MRP2 and is sensitive to various bile acids e.g., taurocholate [126]. MRP2 is highly expressed in liver and its activity significantly changes in liver pathology [76,126]. Hepatic MRP2 expression is increased in liver and intestinal ischemia-reperfusion, colitis, liver regeneration, cholestasis, endotoxin exposure, inflammation, acetaminophen and carbon tetrachloride toxicity, exposure to isoflurane, carbamazepine, taurine, tamoxifen, bile-acid treatment, concomitant chronic renal diseases [126]. It decreases in chronic ethanol consumption, methotrexate, cyclosporine A and sirolimus consumption [126]. MRP1 expression enhances in cholestasis, hepatitis C virus infection, endotoxin exposure, hemolysis, liver cancers and tumors, liver regeneration, and oxidative stress [126]. Low serum folate levels have been observed in patients with obesity and diabetes which characteristically are associated with non-alcoholic fatty liver disease (NAFLD) [182,183,184]. However, the results in patients with NAFLD are somewhat contradictory [161,185]. In rodents, low folates level perturbs one-carbon metabolism and may be associated with development of NAFLD [186]. Significant correlation between low levels of folate and vitamin B_12_ and histological severity of non-alcoholic steatohepatitis was found in one study [187]. In an animal model of NAFLD (mice fed a high-fat diet) folic acid supplementation reduced the number of inflammatory foci and lipid vacuoles in the liver which was correlated with reduced expression of pro-inflammatory cytokines [186]. However, in humans, six months of therapy with folic acid 1 mg/day did not lead to a significant biochemical improvement in patients with NASH [185]. Meta-analysis revealed that NAFLD was associated with an increased risk of hyperhomocysteinemia, even in the absence of difference in folate level and vitamin B_12_ level between NAFLD subjects and healthy controls, that is why it seems to be actual to control folate level in NAFLD [188]. In patients with arterial hypertension without known liver disease, daily enalapril combined with 0.8 mg of folic acid showed a beneficial effect on serum ALT level than with treatment with enalapril alone [189].

Kidneys are rich in folate receptors RFC expressed to the basolateral membrane and FRα abundantly located to the apical surface of canalicular cells [190]. Urinary excretion represents the major route of folate and antifolates elimination. OAT-K1 expressed predominantly in renal straight tubules play an important role in this process. Folate absorption from the proximal tubule may counteract the development of deficiency [49]. In advanced chronic kidney disease folate metabolism must be considered due to serious homeostatic disorders altering cellular functions. In end stage kidney disease, an insignificant proportion of patients, diminished serum folate concentration was shown due to malnutrition, low uptake and incorporation of 5-methyl-THF into cellular cycles [191,192].

A few studies have associated low folate level with *Helicobacter pylori* infection. A decreased folate absorption may be a consequence of an increment in pH and/or low vitamin C concentration in gastric juice which is observed in *H. pylori*—infection [193]. National Health and Nutrition Examination Surveys (NHANES 1999–2000) cross-sectional data among adults (*n* = 3,055) raised a problem of indirect pathways leading to impaired folate metabolism (through the antioxidant status) [194]. Another paper discussing the probable association of *H. pylori* infection with coronary plaques formation underlines the patients had significantly lower vitamin B_12_ and higher homocysteine level, although their folic acid concentration showed (8.9 ± 3.2 vs. 10.0 ± 3.6 ng/mL; *p* = 0.171) no significant difference with controls [195]. Another study shows near similar results [195]. In children, no significant difference in folate levels between *H. pylori*-positive and -negative patients was found [196]. 

## 9. Risks Associated with Folate Deficiency 

### 9.1. Macrocytic Anemia, Mucositis, Infertility, Muscular Weakness

In folate deficiency actively proliferating tissues are affected—mostly bone marrow with development of macrocytic anemia, mucosal sores, infertility in males [197,198]. Muscle weakness and walking difficulty are also typical features of folate deficiency, which induces proliferation inhibition and cellular senescence in C2C12 myoblasts [199].

### 9.2. Cardiovascular Disease 

In the 1990th the problem of hyperhomocysteinemia as an independent risk factor for atherosclerosis has been raised [200,201,202].

Folic acid is an important dietary determinant of homocysteine level (Figure 3). Supplementation with 0.5–5.0 mg/day could lower serum homocysteine level about a quarter and the risk of ischemic heart disease and stroke by 11% and 19%, respectively [203,204]. The results of double-blind placebo-controlled trials assessing the role of folic acid supplementation in the prevention of cardiovascular events are somewhere contradictory. In placebo-controlled double-blind Aspirin/Folate Polyp Prevention Study supplementation with folic acid 1 mg/day (the separate group included participants received low-dose aspirin) did not show the difference in the incidence of cardiovascular disease and all-cause mortality between the intervention and placebo groups [205]. In the placebo-controlled double-blind Vitamins to Prevent Stroke Study the patients with a known history of stroke were randomized to B-complex vitamins and folic acid supplementation or a placebo. No significant difference in the incidence of stroke, myocardial infarction or vascular-related death was found between intervention and control groups [206]. Meta-analysis of 12 randomized controlled trials, totally enrolled 47,523 patients, showed no significant difference in all-cause mortality, cardiovascular mortality and risk of coronary heart disease, despite the decreased risk of stroke (RR = 0.85, 95% CI = 0.77–0.94, *P*_heterogeneity_ = 0.347, *I^2^* = 10.6%) [207]. 

Elevated homocysteine levels (22.9 ± 3.5 vs 9.0 ± 2.3 μmol/L in controls, *p* < 0.001) and low folate levels (6.7 ± 5.0 ng/mL and 9.0 ± 4.4 ng/mL in controls, *p* < 0.05) were significantly associated with arterial hypertension [208]. In contrast, the large China Stroke Primary Prevention Trial enrolled 20,000 patients with primary hypertension and known MTHFR C677T genotype and without history of myocardial infarction or stroke, clarifies the potential benefits of folic acid supplementation. The participants were randomized to treatment with enalapril alone or enalapril combined with folic acid supplementation. At a 4.5 years-median follow-up the group taking enalapril with folic acid showed a greater reduction in the incidence of ischemic stroke and composite cardiovascular events with more pronounced results in patients with low baseline folate level [209]. It can be concluded from this study that folic acid can be recommended for stroke prevention in the regions with high folate deficiency prevalence and without appropriate food fortification [209].

### 9.3. Neurological Problems

Folic acid is essential for early neurodevelopment and is known to protect against neural tube defects, mostly spina bifida; the neural tube closes approximately six weeks after implantation. National health agencies worldwide recommend for women of childbearing age to take 0.4–1 mg/day of supplementary folic acid to reduce the risk of neural tube defects [157]. FRα plays an important role in glial cell differentiation [83]. CNS folate transport is regulated mostly by choroid plexus. Serum 5-methyl-THF is uptaken by FRα while RCF located on the basolateral membrane on choroid epithelial cells is involved in its transferring to glia (Figure 2) [116,210]. Folate deficiency contributing to neurological manifestation in adults is not well studied, while most papers are focused rather on the problem of cobalamin deficiency. Nevertheless, folate is very important cobalamin partner in methionine, purine and myelin synthesis. Concerns about masking cobalamin deficiency by folic acid could be lessened by adding 1 mg of cobalamin to folic acid supplements [201]. Lack of S-adenosylmethionine if CNS may be followed by decreased methylation of norepinephrine and acetylserotonin and low formation on epinephrine and melatonin, respectively [145]. Hyperhomocysteinemia typical of folic deficiency may also lead to CNS impairment, probably though the ischemic mechanism and synaptic dysfunction that, in its turn, develops as a result of endoplasmic reticulum stress and excessive glutamatergic receptors activation, leading to excitotoxicity [211]. In contrast to other tissues, there is no alternative way of homocysteine remethylating and maintaining of S-adenosylmethionine synthesis (from betaine) in CNS. This fact may explain early manifestations of neurological signs in folate deficiency. Glial cells are most sensitive to lack of methylation [212]. Hypomethylation and abnormal membrane fluidity affects the function of serotonin and dopamine transporters and the structure of dopamine receptors [213,214]. The origin of cognitive, mental and other psycho-neurological disorders in the absence of typical of folate deficiency macrocytosis sometimes gets the wrong interpretation as “cryptogenic”, “reactive”, “dyscirculatory” [215]. THF is involved in formation of glycine necessary for CNS function (Figure 3) [216]. Folic acid inhibits expression of matrix metalloproteases-2, -9 playing role in neuropathic pain after spinal cord injury in rats [217].

### 9.4. Cancer 

Some animal models demonstrate folate-deficient diet would induce genotoxicity and some data from clinical studies shows folate supplementation may reduce the risk of progression of mucosal precancerous lesions [218]. In the study included patients with recurrent mild or moderate laryngeal dysplasia effect of prophylactic treatment with 400 mg folic acid/day for 6 months was assessed. 58% showed clinically evident regression of leukoplakia and 25%—reduced volume of the affected area. In the control group, 67% exhibited no change, and in 25% there was disease progression with suspected malignant transformation. Despite the small number of study participants and absence of placebo control, its considered design and clear results indicate perspectivity of this approach [219]. In the model of skin tumorigenesis with 7,12-dimethylbenz(a)anthracene folic acid decreased the cancerogenic potential as indicated by decreased epidermal thickness and cell count, expression enzymes indicating cell proliferation, lipid peroxidation and reduced glutathione [220]. In meta-analysis of nine studies assessing the risk of head and neck squamous cell carcinoma, depending on the level of folate intake, the pooled OR of the malignancy in the highest versus lowest doses was 0.505 (95% CI 0.387–0.623). Linearity analysis indicated that with increased 100 μg/d folate intake, the risk of head and neck squamous cell cancer decreased 4.3% degree (OR 0.957, 95% CI 0.935–0.980) [221]. A particular issue is risk of malignancy in diabetes mellitus. In type 2 diabetes mellitus (T2DM), an elevated baseline of genomic instability was found. High blood glucose and low blood folate are prevalent in T2DM, we hypothesized that high glucose may work with low folate to induce genomic instability. To assess the potential role of folic acid in tumorigenesis in type 2 diabetes mellitus genotoxic effects of high sugars was studied in cell lines NCM460, CCD841, and L02 (over a course of 7 days by the cytokinesis block micronucleus assay). High sugars possessed genotoxicity under folate depletion condition which didn’t manifest under folate repletion. These results show the importance of folate level control in the prevention of gastrointestinal neoplasia in diabetes mellitus [222].

## 10. Antifolates

Antifolates are used for the treatment of malignancy (acute myeloid leukemia, osteosarcoma, lung cancer), chronic inflammatory disorders (rheumatoid arthritis, inflammatory bowel disorders), and bacterial and parasitic infections. “Classical” antifolates (e.g., methotrexate, pemetrexed, pralatrexate, raltitrexed) have a pterin core and glutamate extension, and utilize RFC as the major route of intracellular efflux [144]. Intracellular polyglutamylation is necessary for their retention and achievement of effective concentration for inhibition of folate-dependent enzymes [223]. Antifolate polyglutamylates cannot be extruded from the cell via ATP-dependent efflux transporters [118,122,128,175]. Classical antifolates exhibit antitumor activity affecting metabolic cycles essential for cellular replication—inhibiting dihydrofolate reductase (methotrexate, pralatrexate, pyrimethamine, etc.), thymidylate synthetase (pemetrexed, raltitrexed), glycinamide ribonucleotide formyl transferase (pemetrexed) [135,224].

“Nonclassical” propargyl-linked antifolates (e.g., trimethoprim) enter the microbial and parasitic cells by passive diffusion, are not converted to the polyglutamates and therefore are not significantly retained [223]. Nonclassical antifolates inhibit dihydrofolate reductase providing antibacterial activity [223]. New series of antibiotics, propargyl-linked diaminopyrimidines having the core structure similar to that of trimethoprim is under development [224].

### Resistance to Methotrexate and Other Antifolates and Their Toxicity 

Cells can acquire resistance to antifolates related to reduction of intracellular influx, reduced intracellular polyglutamylation, changes in target enzymes activity, and also P-glycoprotein-type resistance. Resistance to antifolates results in alleviated response to treatment.

RCF is the most important route for classical antifolates, while FR is essential for the uptake of inhibitors propargyl-linked antifolates (such as CB3717, ICI-198) and the inhibitor of purine synthesis, 5,10-dideazatetrahydrofolate [50]. Pemetrexed is characterized by near equal affinity both for PCFT and RFC at physiological pH [107]. As PCFT becomes active in more acidic tumor environment, new generations of 6-substituted pyrrolopyrimidine compounds with selective PCFT-mediated transport and effective in malignant mesothelioma and non-small cell lung cancer is under development [144].

In tumors and cultured neoplastic cells, resistance to classical antifolates (e.g., methotrexate) associated with decreased RFC function may develop due to negative regulation of RFC activity or point mutations in RFC gene. The latter may lead to splicing of non-functional polypeptide or polypeptide having another substrate specificity [49]. Methotrexate resistance relevant to decreased FR expression also was described [49]. Although hydrophilic methotrexate enters the cell mainly using folate transporters, passive diffusion becomes the mode of drug uptake in the case of their inactivity [225]. 

Given the fact that folate receptors are associated with the cellular folylpoly-γ-glutamate compartment, the degree of methotrexate polyglutamination, in this case, is not well understood. 

Loss of function of ATP-dependent cytoplasmic and mitochondrial enzyme folylpoly-γ-glutamate synthetase leads to a dramatic reduction of intracellular methotrexate polyglutamate level and absence of their intracellular accumulation [133].

Mitochondrial NADH dehydrogenase 1 beta subcomplex subunit 7 (syn—protein SQM1) transferring electrons from NADH to the respiratory chain may affect methotrexate transport. Expression of SQM1 was shown to be reduced in cell lines resistant to methotrexate [226].

Initial folate status and receptors activity are the important prognostic factors for successful and safe methotrexate treatment [49]. Worsening of methotrexate toxicity might be explained by increase of the density of folate receptors in normal cells as a consequence of previous long-term folate restriction [49]. Short term folate deprivation may lead to decrease synthesis of RFC and γ-glutamate hydrolase mRNA consistent with fall in methotrexate influx and effectiveness [30,34,48]. In contrast, cellular folate deprivation may be associated with higher FRs expression and with inhibition of ATP-driven folate export pumps excluding BCRP [34,50,129]. Folic acid supplementation in the course of antifolate therapy may overcome receptors rearrangements and methotrexate toxicity, but due to competition with antifolate action may also diminish its antitumor activity [49]. In folic acid supplementation, THF formation for consequent regulatory genes methylation is required that is decreased due to inhibition of dihydrofolate reductase under the influence of antifolate. Folinic acid (leucovorin) is a synthetic vitamer of folic acid having the structure of formyl-THF that could be converted to 5,10-methylene-THF and to 5-methyl-THF without dihydrofolate reductase and has also maximal RFC affinity. Folinic acid supplementation results in a significant reduction in common side effects of methotrexate due to control of folate receptors expression [174].

Polymorphism in the RFC gene and also in genes controlling intracellular folate and antifolate transformations may also be involved in antifolate toxicity due to excessive accumulation in the cells [151]. In Chinese patients with non-small cell lung cancer MTHFR gene *polymorphism* C665T was found to be significantly associated with hematological pemetrexed adverse reactions (*p* = 0.0079, OR = 3.566) while 5-aminoimidazole-4-carboxamide-ribonucleotide formyltransferase gene polymorphism T102C and gamma-glutamyl hydrolase gene polymorphism G91T were associated with both adverse reactions and therapeutic effects. Analysis for these polymorphisms may help to predict the course of treatment [227].

P-glycoprotein and MRPs inhibitors (e.g., medications, such as reserpine, verapamil, and quinidine, flavonoids, coumarins, terpenoids, alkaloids and saponins) can partially reduce the efflux of folates and methotrexate increasing the risk of its toxic action [126,127]. In turn, in tumor cells, expression of P-glycoprotein results in reduction of intracellular antifolate concentrations [127].

Folinic acid-stimulated tubular secretion of methotrexate via OAT-K1 and OAT-K2 is very important in prevention of methotrexate renal toxicity. Decreased expression of these transporters may attribute to longer methotrexate exposure and low effectiveness of antitoxic folinic supplementation [130].

## 11. Conclusions

Folate compounds act as cofactors in the one-carbon metabolism, which is one of the most fundamental for cellular life. We need to accept that our understanding of the regulation of folate uptake and transformations is so far limited. We know a lot about the structure and operation of folate carriers, and the according genes were sequenced. However, little is known about the local tissue regulation of folate transporters and alternative splicing of their genes. Despite folate abundance and high content in many foods, the prevalence of folate deficiency in the population is high enough and remain to be a real problem. Gathered evidence of serious problems associated with an increased folate consumption, particularly those associated with excessive DNA methylation, indicate the prudent approach for food fortification is needed. It would be safer and more effective to recommend additional folate administration to certain population groups, e.g., women of reproductive age and the elderly, applying the concept of “window of opportunity”. To follow this principle, it is necessary to perform regular analysis of scientific and data continue the heated discussions.

## Figures and Tables

**Table 1:** Folic Acid Content in Foods [According to the National Institutes of Health, Food Supplements Administration [11].

Food	Daily Value (%)
Beef liver, braised, 3 ounces	54
Spinach, boiled, ½ cup	33
Black-eyed peas, boiled, ½ cup	26
Breakfast cereals, fortified with 25% of the DV	25
Rice, white, medium-grain, cooked, ½ cup	22
Asparagus, boiled, 4 spears	22
Brussels sprouts, frozen, boiled, ½ cup	20
Spaghetti, cooked, enriched, ½ cup	19
Lettuce, romaine, shredded, 1 cup	16
Avocado, raw, sliced, ½ cup	15
Spinach, raw, 1 cup	15
Broccoli, chopped, frozen, cooked, ½ cup	13
Mustard greens, chopped, frozen, boiled, ½ cup	13
Bread, white, 1 slice *	13
Green peas, frozen, boiled, ½ cup	12
Kidney beans, canned, ½ cup	12
Wheat germ, 2 tablespoons	10
Tomato juice, canned, ¾ cup	9
Crab, Dungeness, 3 ounces	9
Orange juice, ¾ cup	9

* Fortified with folic acid as part of the folate fortification program.

**Table 2:** Recommended levels of folic acid intake per day depending on age.

Age, Years	Adequate Intake, µg/Day	Tolerable Upper Intake Level, µg/Day
1–3	150	300
4–8	200	400
9–13	300	600
14–18	400	800
19+ *	400–600	1000

* including pregnancy and lactation.

**Table 3:** Determination of folate status using macrocytic anemia as hematologic indicator [150,173].

Serum/Plasma Folate ng/mL (nmol/L)	Ber Blood Cells Folate ng/mL (nmol/L)	Interpretation
>20 (>45.3)		Increased
6−20 (13.5−45.3)		Normal
3−5.9 (6.8−13.4)		Probable deficiency
<3 (<6.8)	<100 (<226.5)	Deficiency

**Table 4:** Thresholds for determining folate deficiency using homocysteine concentrations as an indicator [173].

Parameter	Homocysteine Level ng/mL (nmol/L)
Serum/plasma folate	<4 (<10)
Red blood cell folate	<151 (<340)

## Data Availability

This is a review paper that collected from public data listed in the “Reference” and from open accessible web-sources (all web-link were valid upon this Review paper has been published).
